# SNARE zippering

**DOI:** 10.1042/BSR20160004

**Published:** 2016-05-06

**Authors:** Xiaochu Lou, Yeon-Kyun Shin

**Affiliations:** * Roy J. Carver Department of Biochemistry, Biophysics and Molecular Biology, Iowa State University, Ames, IA 50011, U.S.A.

**Keywords:** coiled coil, complexin, four-helix bundle, Munc18-1, nanodisc, SNARE, synaptotagmin 1

## Abstract

SNARE (soluble *N*-ethylmaleimide-sensitive factor attachment protein receptor) proteins are a highly conserved set of membrane-associated proteins that mediate intracellular membrane fusion. Cognate SNAREs from two separate membranes zipper to facilitate membrane apposition and fusion. Though the stable post-fusion conformation of SNARE complex has been extensively studied with biochemical and biophysical means, the pathway of SNARE zippering has been elusive. In this review, we describe some recent progress in understanding the pathway of SNARE zippering. We particularly focus on the half-zippered intermediate, which is most likely to serve as the main point of regulation by the auxiliary factors.

## INTRODUCTION

Many vital life processes in eukaryotic cells, such as trafficking of proteins or membranes and secretion of hormones or neurotransmitters, require membrane fusion. Intracellular membrane fusion must happen in a specific and regulated manner. For this, highly specialized proteins called ‘fusogens’ mediate the merging of two otherwise stable membranes to a single bilayer. It is now established that widely conserved soluble *N*-ethylmaleimide-sensitive factor attachment protein receptor (SNARE) proteins are the primary fusogen, responsible for nearly all intracellular membrane fusion [1–3].

Since the discovery of SNAREs in late 80s through early 90s significant progress has been made towards understanding the mechanism by which SNAREs drive membrane fusion. Vesicle-anchored SNARE (v-SNARE) associates with the target membrane-anchored SNARE (t-SNARE) to form a SNARE complex. More precisely, individual SNAREs contain SNARE motifs that are essentially coiled coil sequences of 60–70 residues [4,5]. Cognate coiled–coiled interactions between v- and t-SNAREs are the basis for SNARE complex formation [6,7]. The complex is however believed to assemble in multiple steps, each of which may be mechanically-coupled to a membrane remodelling step [8]. Eventually, the SNARE complex ends up a four-stranded coiled coil [9–14]. This post-fusion conformation has been extensively studied with biochemical and biophysical means.

In contrast, the pathway of SNARE complex formation has been elusive. Thus, the mechanistic details of how the SNARE conformational changes are coupled to membrane remodelling steps are poorly understood. Furthermore, the regulatory interventions of auxiliary factors on SNARE complex formation are not well understood (for reviews see ref. [2,15–18]). However, it has long been speculated that SNAREs might zipper, from the membrane distal N-terminal region towards the membrane proximal C-terminal region [19]. Previously, several research groups have made progress in trapping the partially-zippered intermediate, independently using the advanced biophysical methods [20–24]. These discoveries are major because the results shed lights on to the mechanism of SNARE zippering. The outcomes open up exciting possibilities of studying the regulation of SNARE zippering as mechanisms to control intracellular membrane fusion.

In this review, we describe some recent progress in understanding SNARE zippering and the characterization of the half-zippered intermediate. Additionally, although the data are limited at this early stage we discuss how the half-zippered intermediate might interact with the auxiliary factors to regulate vesicle fusion, particularly for Ca^2+^-triggered membrane fusion at the synapse.

## PRELUDE TO SNARE ZIPPERING

**Figure 1:**
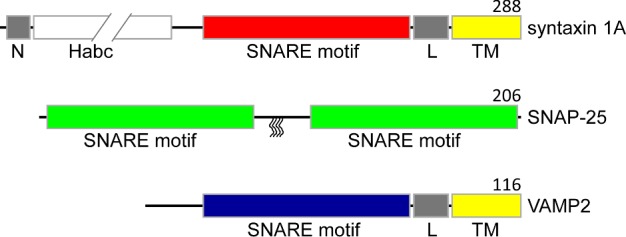
Primary structures of all three SNAREs in a bar representation, showing different domains SNARE motifs are coloured in red (syntaxin 1A), green (SNAP-25) and blue (VAMP2). In syntaxin 1A and VAMP2, the TM regions are labelled ‘TM’ and coloured in yellow whereas the linker regions are labelled ‘L’ and coloured in grey. The Habc domain (white) of syntaxin 1A is depicted to be broken to indicate the longer length than the SNARE motif (red). The syntaxin 1A N-terminal peptide is labelled ‘N’ (grey). In SNAP-25, the four palmitoylated cysteines are denoted as wiggly lines. Numbers on the right above the diagrams indicate the length of the proteins.

One of the best characterized SNARE families is the neuronal one involved in synaptic vesicle fusion, which is required for neurotransmitter release into the synapse cleft. We will focus on the structure and the function of neuronal SNAREs throughout the review unless otherwise noted. In the present study, vesicle-associates membrane protein 2 or synaptobrevin 2 (VAMP2) is v-SNARE whereas syntaxin 1A and synaptosomal-associated protein of 25 kDa (SNAP-25) are two t-SNARE entities (Figure 1). These neuronal SNAREs were first individually identified in the nervous system [25–29] and later identified together as a complex, the soluble NSF-attachment protein (SNAP) receptor [30]. VAMP2 and syntaxin 1A both contain one SNARE motif each connected to the C-terminal single transmembrane (TM) helix by a short linker regions whereas SNAP-25 contains two SNARE motifs and is attached to plasma membrane by lipid anchors [31,32]. The individual SNARE proteins are partially or fully unstructured as monomers [24,33,34] although there have been some debates if the SNARE motif of VAMP2 interacts with the vesicle membrane [35,36].

Prior to the interaction with VAMP2, t-SNAREs, syntaxin 1A and SNAP-25 are believed to form a 1:1 binary complex which serves as the receptor for v-SNARE for vesicle docking and fusion [37]. The structure of the 1:1 binary complex of yeast SNAREs was characterized by NMR and it was found that the N-terminal region is well-structured whereas the C-terminal region is frayed [19]. For neuronal SNAREs, syntaxin 1A and SNAP-25 prefer a non-functional 2:1 complex [38,39] *in vitro* instead of the functional 1:1 complex, making it difficult for the structural characterization. However, based on the structure of the 2:1 complex and others [38,39], one might speculate that the 1:1 complex could form an extended three-stranded coiled coil [40] although it remains to be verified experimentally.

## EPILOGUE TO SNARE ZIPPERING: THE ALL PARALLEL FOUR-STRANDED COILED COIL

Ideally, it would be best if one could follow the process of SNARE zippering in the chasm of two membranes whereas v- and t-SNAREs are anchored to apposing membranes. However, this is a tall order and alternatively, one could study the interaction between soluble recombinant SNARE motifs out of context with membranes although there is a serious caveat with this approach that we will discuss later.

**Figure 2:**
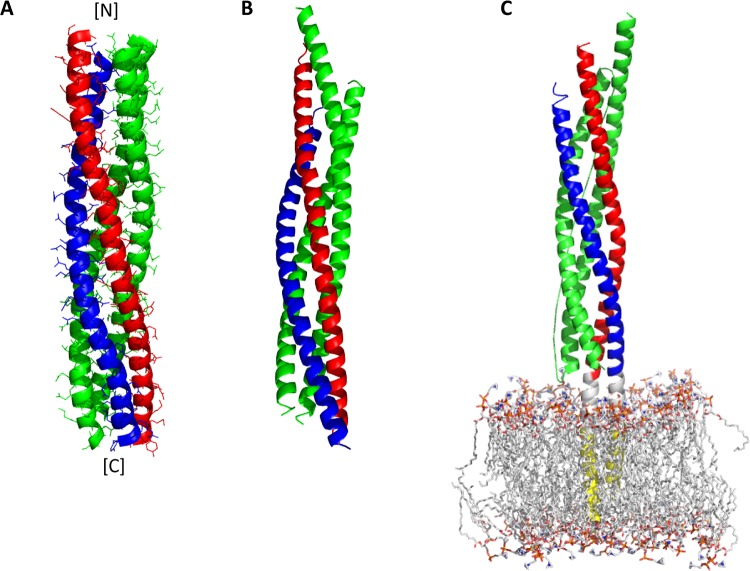
Structures of four-helix bundles (**A**) A structural model of the SNARE core (blue, VAMP2; red, syntaxin 1A; green, SNAP-25) generated from the distance information obtained by EPR. (**B**) A backbone cartoon drawing of the SNARE core determined by X-ray. (**C**) A model of the *cis*-SNARE complex inserted into the POPC (1-palmitoyl-2-oleoyl-*sn*-glycero-3-phosphocholine) membrane. The letters N and C in square brackets indicate the N- and C-terminus of the protein respectively. The figures are prepared with PyMOL [65] using the PDB files 2UB0 (A), 1SFC (B) and 3HD7 (C) [9–11].

The early EM and the FRET  works suggested that syntaxin 1A and VAMP2 align parallel in the SNARE complex, consistent with the general idea that SNARE complex formation would bring about the close apposition of two membranes [41,42]. Later, EPR and X-ray crystallography showed that SNARE motifs assemble as an all parallel four-stranded coiled coil [9,10] (Figure 2). The SNARE core contains 15 layers of interacting hydrophobic side chains, and right at the centre there is a central ionic layer consisting of one arginine (R) residue from VAMP2 and three glutamine (Q) residues from syntaxin 1A and SNAP-25. Accordingly, SNARE motifs are often classified into R, Qa, Qb and Qc types [43,44]. This highly conserved feature appears to play an important role in SNARE zippering (see below). Recently, the X-ray structure of the neuronal SNARE complex that includes the TM regions of both syntaxin 1A and VAMP2 has been determined [11]. The structure showed that both syntaxin 1A and VAMP2 extend their helical structures of SNARE motifs through the TM helices [11] (Figure 2C). Apparently, these structures are most likely to represent the post-fusion SNARE conformation.

## SNARE ZIPPERING AND THE DISCOVERY OF THE HALF-ZIPPERED SNARE INTERMEDIATE

It has long been speculated that the SNARE complex assembles in a zipper-like fashion, proceeding from the N-terminal region towards the C-terminal region, which would progressively narrow the gap between two membranes. Consistently, there is evidence that SNARE complex formation takes place in multiple steps. Firstly, because the SNARE core is stabilized by the hydrophobic layers, the disrupting mutations at the C-terminal hydrophobic layers affect the fast phase of exocytosis *in vivo*. These mutations result in two-step thermal unfolding *in vitro* [45]. Secondly, the force compared with distance measurement using a surface force apparatus (SFA) reveals that the SNARE complex assembles through a series of intermediates [46]. Thirdly, a partially-zippered SNARE complex with a frayed C-terminal region was trapped by intercalating a small hydrophobic molecule myricetin into the SNARE core [20].

More direct characterization of the partially-zippered intermediate in a single molecule level was achieved using high-resolution optical tweezers and also, independently, using magnetic tweezers [21,22]. These experiments were made possible by attaching one handle at the C-terminal end of v-SNARE and the other handle at that of t-SNARE respectively. Optical tweezers reveals that SNARE unzipping proceeds through three distinct stages with two transitions, the first occurring near the juxtamembrane region and the second at the C-terminal half. The half-zippered intermediate could be stabilized by external force and can release ∼36 *k*_B_*T* by transitioning to the fully zippered state [21]. On the other hand, the magnetic tweezers reveals that single SNARE complex can be unzipped with 34 pN force and rezipping is achieved by lowering the force below 11 pN. Here, a half-zippered state could be stably held under the constant force of 11 pN. Thus, the results detail the energy landscape of SNARE zippering [22]. The valuable information from these studies would eventually help correlating the mechanics of SNARE zippering and the energetics of membrane fusion.

**Figure 3:**
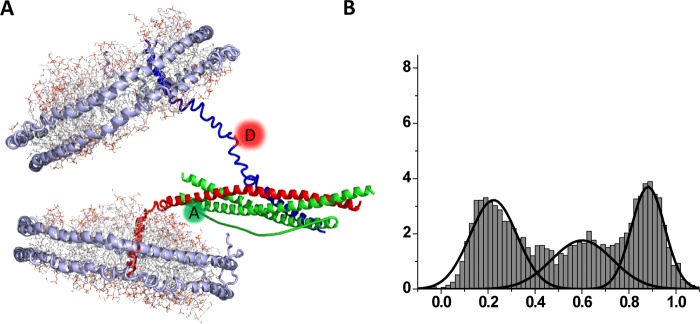
A structural model of the half-zippered intermediate (**A**) *Trans*-SNAREpin representing the fusogenic pre-fusion intermediate trapped between two nanodisc membrane patches. smFRET between the donor and the acceptor dyes attached to v- and t-SNAREs respectively reports SNARE zippering. (**B**) Distribution of the FRET efficiency between fluorescent dye-labelled C-terminal positions on VAMP-2 and syntaxin 1A respectively. The low FRET population represents the half-zippered species whereas the high FRET population reflects the fully zippered species [23].

Furthermore, some structural details of the partially-zippered SNARE intermediate have been obtained with EPR using a ‘nanodisc sandwich’ [23]. Experimentally, two nanodiscs which bear single VAMP2 and single t-SNARE respectively are prepared and SNARE complex formation is allowed between two nanodiscs, creating a nanodisc sandwich that harbours a single *trans*-SNARE complex in the middle (Figure 3A). Due to the rigid structure of nanodiscs membrane fusion does not occur, and the transient SNARE intermediate is captured and studied with SDSL (site-directed spin labelling) EPR as well as single molecule FRET (smFRET) [23]. The nitroxide-scanning EPR study shows that an apparent structural hinge for SNARE zippering is located exactly at the ^1^RQ^3^ ionic layer, potentially revealing the structural role of this highly conserved feature. Furthermore, smFRET with the acceptor and donor pairs near the C-terminal ends of v- and t-SNAREs respectively show that the half-zippered intermediate is energetically balanced with the fully zippered state, exhibiting two well-defined low FRET and high FRET populations (Figure 3B).

## BLESSINGS AND CURSES OF THE FOUR-STRANDED COILED COIL

The determination of the four-stranded coiled coil structure has been clearly one of the most important blessings towards understanding the mechanism of intracellular membrane fusion. Very interestingly, the SNARE core shares many important structural features with viral fusogens [47], strongly arguing for the possibility that common biophysical and biochemical principles do exist and are shared by many biological membrane fusion systems, if not all. However, what distinguishes SNARE-dependent membrane fusion from other membrane fusion systems is the sophistication in its regulation.

One of the remarkable features of synaptic membrane fusion is its capacity to synchronize fusion of nearly all vesicles in the readily releasable pool (RRP) to the presynaptic membrane in less than 1 ms upon Ca^2+^ influx [48]. It is believed that such tight regulation is orchestrated by a series of exquisitely coordinated interactions of auxiliary factors with SNAREs. A major Ca^2+^-sensor synaptotagmin 1 (syt1), a clamping factor complexin (cpx) and a chaperon Munc18-1 are considered as the major regulatory components for the synchronization. According to a current mechanistic model [49–51], membrane fusion is clamped by cpx prior to the Ca^2+^ influx. But upon Ca^2+^ influx, the Ca^2+^-bound syt1 knocks off the cpx clamp from the SNARE complex, which frees the SNARE complex to be able to drive membrane fusion. Now, the important question is whether we could test and verify this mechanistic model structurally.

An easy access to the coiled coil structure of the SNARE core let us attempt to address this question by examining the impact of auxiliary factors to the SNARE core. But the outcomes of this approach have been confusing at best. For example, the structure of the SNARE core bound to cpx reveals that cpx binds to the surface groove on the coiled coil without the anticipated disruption of the SNARE core structure [52]. Furthermore, when VAMP2 SNARE motif is shortened at the C-terminal region, cpx cross-links neighbouring SNARE cores in a zigzag fashion [53], which initiated contested debates in the fields for the biological validity of the structure [54,55]. Likewise, two structural models for the syt1–SNARE core interactions paint very different pictures from each other [56,57]. Thus, it still remains to be seen if these structures truly represent the action of syt1 in triggering vesicle fusion.

## HALF-ZIPPERED SNARE INTERMEDIATE AS A POTENTIAL TARGET OF REGULATION

A caveat of studying the interaction between the SNARE core and the auxiliary factors is that the four-helix bundle may well be the post-fusion conformation. It is more than likely that auxiliary factors interfere with SNARE zippering at early stages to clamp, decelerate or accelerate zippering [17]. Since the half-zippered SNARE intermediate is now accessible [21–23,58], one could explore the possibility that it is indeed the point of the regulation.

This hypothesis could be tested experimentally. For example, with optical or magnetic tweezers one could ask if auxiliary factors affect the distance compared with force relationship for the SNARE core [59]. Although the experiments appear to be straightforward one intrinsic difficulty with these approaches is the absence of membranes, particularly because it is known that syt1 and cpx both interact with the membrane [60,61].

**Figure 4:**
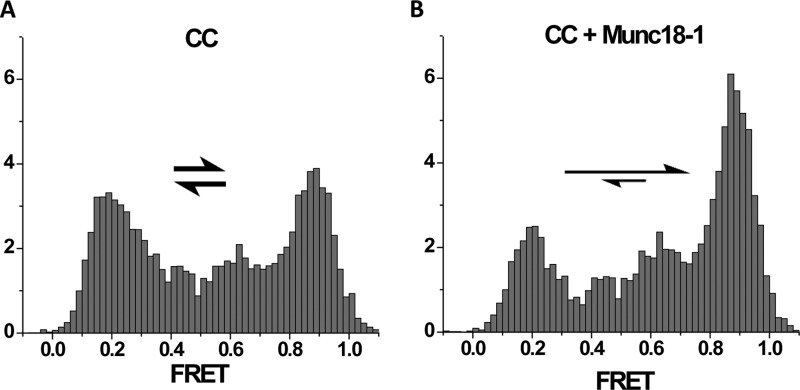
Munc18-1 stimulates SNARE zippering Histograms of the smFRET distribution for the FRET pair attached at the C-terminal region of the *trans*-SNAREpin (CC) trapped inside the nanodisc sandwich [23,62] (see Figure 3). For the SNAREpin low and high FRET peaks are nearly equally populated (**A**). Mun18-1 shifts the equilibrium towards high FRET (**B**).

An alternative but promising approach is to use the nanodisc sandwich that harbours the half-zippered intermediate in the middle [23]. There the half-zippered intermediate is energetically balanced and thus, is in equilibrium with the fully zippered SNARE complex [23,62] (Figure 4A). The conformational changes or the shift of equilibrium induced by the auxiliary proteins may be detected by placing nitroxide probes or fluorescence labels at strategic positions in the SNARE complex. Such efforts are already underway and start to produce some interesting results. For example, Munc18 is shown to stimulate SNARE-dependent membrane fusion [63]. However, the proposition was relied heavily on a simplified *in vitro* proteoliposome fusion assay [64]. Consistent with this notion, smFRET with the FRET dye pair placed at the C-terminal ends of v- and t-SNARE respectively shows the shift of the equilibrium towards fully zippered complex in expense of the half-zippered intermediate [62] (Figure 4B).

For cpx, however, to be consistent with its fusion clamping role we expect that cpx either shifts the equilibrium towards the half-zippered intermediate from the fully zippered complex or it might lock the SNARE intermediate at a yet unknown state that can prevent it from progressing towards the fully zippered state.

Ultimately, structures of the *trans*-SNAREs complexed with individual auxiliary proteins in the nanodisc sandwich must be determined to fully comprehend the mechanisms. EPR or cryo EM may be useful for these challenging tasks.

## SUMMARY

SNARE proteins, which are widely conserved from yeast to human, are the core machinery for intracellular membrane fusion. Vesicle-associated v-SNARE associate with target membrane t-SNARE to drive the fusion of two membranes. There is now sufficient evidence that the SNARE complex assembles in a zipper-like fashion, initially at the membrane distal N-terminal region and subsequently at the membrane proximal C-terminal region. Using single molecule manipulation techniques such as optical and magnetic tweezers, the energy landscape of the multistep folding/unfolding transitions of the SNARE complex have been determined in an unprecedented accuracy. Furthermore, it was found, with EPR that SNARE zippering hinges precisely at the conserved ‘zero’ layer. The half-zippered SNARE intermediate can be trapped in trans between two nanodiscs, where the half-zippered state is in equilibrium with the fully zipped state. This conformational trap inside the nanodisc sandwich provides exciting opportunities to investigate the intervention of auxiliary proteins on to SNARE zippering as means of regulating intracellular membrane fusion such as Ca^2+^-triggered synaptic vesicle fusion.

## Abbreviations

cpx: complexin
smFRET: single molecule FRET
SNAP-25: synaptosomal-associated protein of 25 kDa
SNARE: soluble *N*-ethylmaleimide-sensitive factor attachment protein receptor
syt1: synaptotagmin 1
TM: transmembrane
t-SNARE: target membrane-anchored SNARE
VAMP2: vesicle-associates membrane protein 2
v-SNARE: vesicle-anchored SNARE

## References

[1] Weber T., Zemelman B.V., McNew J.A., Westermann B., Gmachl M., Parlati F., Sollner T.H., Rothman J.E. (1998). SNAREpins: minimal machinery for membrane fusion. Cell.

[2] Sudhof T.C., Rothman J.E. (2009). Membrane fusion: grappling with SNARE and SM proteins. Science.

[3] Wickner W., Schekman R. (2008). Membrane fusion. Nat. Struct. Mol. Biol..

[4] Weimbs T., Low S.H., Chapin S.J., Mostov K.E., Bucher P., Hofmann K. (1997). A conserved domain is present in different families of vesicular fusion proteins: a new superfamily. Proc. Natl. Acad Sci U.S.A..

[5] Weimbs T., Mostov K., Low S.H., Hofmann K. (1998). A model for structural similarity between different SNARE complexes based on sequence relationships. Trends Cell Biol..

[6] Fasshauer D., Otto H., Eliason W.K., Jahn R., Brunger A.T. (1997). Structural changes are associated with soluble N-ethylmaleimide-sensitive fusion protein attachment protein receptor complex formation. J. Biol. Chem..

[7] Poirier M.A., Hao J.C., Malkus P.N., Chan C., Moore M.F., King D.S., Bennett M.K. (1998). Protease resistance of syntaxin.SNAP-25.VAMP complexes. Implications for assembly and structure. J. Biol. Chem..

[8] Hernandez J.M., Stein A., Behrmann E., Riedel D., Cypionka A., Farsi Z., Walla P.J., Raunser S., Jahn R. (2012). Membrane fusion intermediates via directional and full assembly of the SNARE complex. Science.

[9] Poirier M.A., Xiao W., Macosko J.C., Chan C., Shin Y.K., Bennett M.K. (1998). The synaptic SNARE complex is a parallel four-stranded helical bundle. Nat. Struct. Biol..

[10] Sutton R.B., Fasshauer D., Jahn R., Brunger A.T. (1998). Crystal structure of a SNARE complex involved in synaptic exocytosis at 2.4 A resolution. Nature.

[11] Stein A., Weber G., Wahl M.C., Jahn R. (2009). Helical extension of the neuronal SNARE complex into the membrane. Nature.

[12] Strop P., Kaiser S.E., Vrljic M., Brunger A.T. (2008). The structure of the yeast plasma membrane SNARE complex reveals destabilizing water-filled cavities. J. Biol. Chem..

[13] Zwilling D., Cypionka A., Pohl W.H., Fasshauer D., Walla P.J., Wahl M.C., Jahn R. (2007). Early endosomal SNAREs form a structurally conserved SNARE complex and fuse liposomes with multiple topologies. EMBO J..

[14] Antonin W., Fasshauer D., Becker S., Jahn R., Schneider T.R. (2002). Crystal structure of the endosomal SNARE complex reveals common structural principles of all SNAREs. Nat. Struct. Biol..

[15] Jahn R., Fasshauer D. (2012). Molecular machines governing exocytosis of synaptic vesicles. Nature.

[16] Rizo J., Sudhof T.C. (2012). The membrane fusion enigma: SNAREs, Sec 1/Munc18 proteins, and their accomplices–guilty as charged?. Annu. Rev. Cell Dev. Biol..

[17] Sudhof T.C. (2013). Neurotransmitter release: the last millisecond in the life of a synaptic vesicle. Neuron.

[18] Rizo J., Xu J. (2015). The synaptic vesicle release machinery. Annu. Rev. Biophys..

[19] Fiebig K.M., Rice L.M., Pollock E., Brunger A.T. (1999). Folding intermediates of SNARE complex assembly. Nat. Struct. Biol..

[20] Yang Y., Shin J.Y., Oh J.M., Jung C.H., Hwang Y., Kim S., Kim J.S., Yoon K.J., Ryu J.Y., Shin J. (2010). Dissection of SNARE-driven membrane fusion and neuroexocytosis by wedging small hydrophobic molecules into the SNARE zipper. Proc. Natl. Acad. Sci. U.S.A..

[21] Gao Y., Zorman S., Gundersen G., Xi Z., Ma L., Sirinakis G., Rothman J.E., Zhang Y. (2012). Single reconstituted neuronal SNARE complexes zipper in three distinct stages. Science.

[22] Min D., Kim K., Hyeon C., Cho Y.H., Shin Y.K., Yoon T.Y. (2013). Mechanical unzipping and rezipping of a single SNARE complex reveals hysteresis as a force-generating mechanism. Nat. Commun..

[23] Shin J., Lou X., Kweon D.H., Shin Y.K. (2014). Multiple conformations of a single SNAREpin between two nanodisc membranes reveal diverse pre-fusion states. Biochem. J..

[24] Kweon D.H., Kim C.S., Shin Y.K. (2003). Regulation of neuronal SNARE assembly by the membrane. Nat. Struct. Biol..

[25] Bennett M.K., Calakos N., Scheller R.H. (1992). Syntaxin: a synaptic protein implicated in docking of synaptic vesicles at presynaptic active zones. Science.

[26] Oyler G.A., Higgins G.A., Hart R.A., Battenberg E., Billingsley M., Bloom F.E., Wilson M.C. (1989). The identification of a novel synaptosomal-associated protein, SNAP-25, differentially expressed by neuronal subpopulations. J. Cell Biol..

[27] Südhof T.C., Baumert M., Perin M.S., Jahn R. (1989). A synaptic vesicle membrane protein is conserved from mammals to Drosophila. Neuron.

[28] Baumert M., Maycox P.R., Navone F., De Camilli P., Jahn R. (1989). Synaptobrevin: an integral membrane protein of 18,000 daltons present in small synaptic vesicles of rat brain. EMBO J..

[29] Trimble W.S., Cowan D.M., Scheller R.H. (1988). VAMP-1: a synaptic vesicle-associated integral membrane protein. Proc. Natl. Acad. Sci. U.S.A..

[30] Söllner T., Whiteheart S.W., Brunner M., Erdjument-Bromage H., Geromanos S., Tempst P., Rothman J.E. (1993). SNAP receptors implicated in vesicle targeting and fusion. Nature.

[31] Hess D.T., Slater T.M., Wilson M.C., Skene J.H. (1992). The 25 kDa synaptosomal-associated protein SNAP-25 is the major methionine-rich polypeptide in rapid axonal transport and a major substrate for palmitoylation in adult CNS. J. Neurosci..

[32] Sollner T., Bennett M.K., Whiteheart S.W., Scheller R.H., Rothman J.E. (1993). A protein assembly-disassembly pathway *in vitro* that may correspond to sequential steps of synaptic vesicle docking, activation, and fusion. Cell.

[33] Liang B., Kiessling V., Tamm L.K. (2013). Prefusion structure of syntaxin-1A suggests pathway for folding into neuronal trans-SNARE complex fusion intermediate. Proc. Natl. Acad. Sci. U.S.A..

[34] Margittai M., Fasshauer D., Pabst S., Jahn R., Langen R. (2001). Homo- and heterooligomeric SNARE complexes studied by site-directed spin labeling. J. Biol. Chem..

[35] Ellena J.F., Liang B., Wiktor M., Stein A., Cafiso D.S., Jahn R., Tamm L.K. (2009). Dynamic structure of lipid-bound synaptobrevin suggests a nucleation-propagation mechanism for trans-SNARE complex formation. Proc. Natl. Acad. Sci. U.S.A..

[36] Brewer K.D., Li W., Horne B.E., Rizo J. (2011). Reluctance to membrane binding enables accessibility of the synaptobrevin SNARE motif for SNARE complex formation. Proc. Natl. Acad. Sci. U.S.A..

[37] Ma C., Su L., Seven A.B., Xu Y., Rizo J. (2013). Reconstitution of the vital functions of Munc18 and Munc13 in neurotransmitter release. Science.

[38] Xiao W., Poirier M.A., Bennett M.K., Shin Y.K. (2001). The neuronal t-SNARE complex is a parallel four-helix bundle. Nat. Struct. Biol..

[39] Zhang F., Chen Y., Kweon D.H., Kim C.S., Shin Y.K. (2002). The four-helix bundle of the neuronal target membrane SNARE complex is neither disordered in the middle nor uncoiled at the C-terminal region. J. Biol. Chem..

[40] Kim C.S., Kweon D.H., Shin Y.K. (2002). Membrane topologies of neuronal SNARE folding intermediates. Biochemistry.

[41] Hanson P.I., Roth R., Morisaki H., Jahn R., Heuser J.E. (1997). Structure and conformational changes in NSF and its membrane receptor complexes visualized by quick-freeze/deep-etch electron microscopy. Cell.

[42] Lin R.C., Scheller R.H. (1997). Structural organization of the synaptic exocytosis core complex. Neuron.

[43] Jahn R., Scheller R.H. (2006). SNAREs–engines for membrane fusion. Nat. Rev. Mol. Cell Biol..

[44] Fasshauer D., Sutton R.B., Brunger A.T., Jahn R. (1998). Conserved structural features of the synaptic fusion complex: SNARE proteins reclassified as Q- and R-SNAREs. Proc. Natl. Acad. Sci. U.S.A..

[45] Sorensen J.B., Wiederhold K., Muller E.M., Milosevic I., Nagy G., de Groot B.L., Grubmuller H., Fasshauer D. (2006). Sequential N- to C-terminal SNARE complex assembly drives priming and fusion of secretory vesicles. EMBO J..

[46] Li F., Pincet F., Perez E., Eng W.S., Melia T.J., Rothman J.E., Tareste D. (2007). Energetics and dynamics of SNAREpin folding across lipid bilayers. Nat. Struct. Mol. Biol..

[47] Skehel J.J., Wiley D.C. (1998). Coiled coils in both intracellular vesicle and viral membrane fusion. Cell.

[48] Schneggenburger R., Neher E. (2000). Intracellular calcium dependence of transmitter release rates at a fast central synapse. Nature.

[49] Tang J., Maximov A., Shin O.H., Dai H., Rizo J., Sudhof T.C. (2006). A complexin/synaptotagmin 1 switch controls fast synaptic vesicle exocytosis. Cell.

[50] Giraudo C.G., Eng W.S., Melia T.J., Rothman J.E. (2006). A clamping mechanism involved in SNARE-dependent exocytosis. Science.

[51] Schaub J.R., Lu X., Doneske B., Shin Y.K., McNew J.A. (2006). Hemifusion arrest by complexin is relieved by Ca2+-synaptotagmin I. Nat. Struct. Mol. Biol..

[52] Chen X., Tomchick D.R., Kovrigin E., Arac D., Machius M., Sudhof T.C., Rizo J. (2002). Three-dimensional structure of the complexin/SNARE complex. Neuron.

[53] Kummel D., Krishnakumar S.S., Radoff D.T., Li F., Giraudo C.G., Pincet F., Rothman J.E., Reinisch K.M. (2011). Complexin cross-links prefusion SNAREs into a zigzag array. Nat. Struct. Mol. Biol..

[54] Krishnakumar S.S., Li F., Coleman J., Schauder C.M., Kümmel D., Pincet F., Rothman J.E., Reinisch K.M. (2015). Re-visiting the trans insertion model for complexin clamping. Elife.

[55] Trimbuch T., Xu J., Flaherty D., Tomchick D.R., Rizo J., Rosenmund C. (2014). Re-examining how complexin inhibits neurotransmitter release. Elife.

[56] Zhou Q., Lai Y., Bacaj T., Zhao M., Lyubimov A.Y., Uervirojnangkoorn M., Zeldin O.B., Brewster A.S., Sauter N.K., Cohen A.E. (2015). Architecture of the synaptotagmin-SNARE machinery for neuronal exocytosis. Nature.

[57] Brewer K.D., Bacaj T., Cavalli A., Camilloni C., Swarbrick J.D., Liu J., Zhou A., Zhou P., Barlow N., Xu J. (2015). Dynamic binding mode of a synaptotagmin-1-SNARE complex in solution. Nat. Struct. Mol. Biol..

[58] Ma L., Rebane A.A., Yang G., Xi Z., Kang Y., Gao Y., Zhang Y. (2015). Munc18–1-regulated stage-wise SNARE assembly underlying synaptic exocytosis. Elife **4**.

[59] Ryu J.K., Min D., Rah S.H., Kim S.J., Park Y., Kim H., Hyeon C., Kim H.M., Jahn R., Yoon T.Y. (2015). Spring-loaded unraveling of a single SNARE complex by NSF in one round of ATP turnover. Science.

[60] Snead D., Wragg R.T., Dittman J.S., Eliezer D. (2014). Membrane curvature sensing by the C-terminal domain of complexin. Nat. Commun..

[61] Chapman E.R. (2008). How does synaptotagmin trigger neurotransmitter release?. Annu. Rev. Biochem..

[62] Lou X., Shin J., Yang Y., Kim J., Shin Y.K. (2015). Synaptotagmin-1 is an antagonist for Munc18–1 in SNARE zippering. J. Biol. Chem..

[63] Shen J., Tareste D.C., Paumet F., Rothman J.E., Melia T.J. (2007). Selective activation of cognate SNAREpins by Sec 1/Munc18 proteins. Cell.

[64] Shin Y.K. (2013). Two gigs of Munc18 in membrane fusion. Proc. Natl. Acad. Sci. U.S.A..

[65] PyMOL (2010).

